# Baseline characteristics and predictors for early implantation of vagus nerve stimulation therapy in people with drug‐resistant epilepsy: Observations from an international prospective outcomes registry (CORE‐VNS)

**DOI:** 10.1002/epi4.13015

**Published:** 2024-08-24

**Authors:** Patrick Kwan, Massimiliano Boffini, Firas Fahoum, Riëm El Tahry, Terence J. O'Brien, Karen Keough, Jane Boggs, Hadassa Goldberg‐Stern, Francesca Beraldi, Gaia Giannicola, Ying‐Chieh Lee, Arjune Sen, CORE‐VNS Registry Group

**Affiliations:** ^1^ The Alfred Hospital Monash University Melbourne Victoria Australia; ^2^ The Royal Melbourne Hospital University of Melbourne Melbourne Victoria Australia; ^3^ LivaNova PLC London UK; ^4^ Tel Aviv Sourasky Medical Center and Tel Aviv University, Neurological Institute Tel Aviv Israel; ^5^ Centre for Refractory Epilepsy Cliniques Universitaires Saint‐Luc Brussels Belgium; ^6^ Child Neurology Consultants of Austin Austin Texas USA; ^7^ Comprehensive Epilepsy Center Wake Forest University Winston‐Salem North Carolina USA; ^8^ Institute of Pediatric Neurology Schneider Children's Medical Center of Israel Petah Tiqva Israel; ^9^ Oxford Epilepsy Research Group John Radcliffe Hospital Oxford UK

**Keywords:** baseline, CORE‐VNS, demographics, drug‐resistant epilepsy, vagus nerve stimulation

## Abstract

**Objective:**

Vagus nerve stimulation (VNS) Therapy is routinely indicated for people with drug‐resistant epilepsy (DRE). We analyzed the baseline characteristics of individuals receiving the recently released VNS models and identified factors associated with early or late implantation.

**Methods:**

The Comprehensive Outcomes Registry of subjects with Epilepsy (CORE‐VNS), a prospective observational study evaluating the clinical and psychosocial outcomes of VNS Therapy®, is following participants for up to 60 months after VNS implantation. In this analysis, we used Cox proportional hazards model to identify baseline characteristics associated with the time from diagnosis to first implantation.

**Results:**

Of the 819 enrolled, 792 (96.7%) participants implanted with a VNS device were evaluated. 529 (64.6%) underwent the first implantation and 263 (32.1%) a re‐implantation. Participants' median age at first implant was 24 years; 492 (62.1%) were ≥18 years old and 166 (20.3%) were < 12 years old. The average number of failed ASMs prior to VNS implantation was 7.1, and 145 (17.7%) had undergone previous epilepsy‐related surgery. Epilepsy was classified as focal in 47.7% of participants, generalized in 16.1% and combined focal and generalized in 34.2%. Many of the participants (40.9%) had epilepsy of unknown etiology. The median time from diagnosis to first implantation was 10.33 years and was significantly shorter in participants with combined focal and generalized epilepsy compared to those with focal epilepsy alone, and in participants with genetic and immune epilepsy compared to those with unknown etiologies.

**Significance:**

In people with DRE, VNS Therapy is provided after multiple failures of ASMs and after failure of epilepsy surgery in one in six individuals. Time from diagnosis to first implantation is associated with epilepsy type and etiology, likely reflecting variable treatment pathways. Clearer guidelines on when and how non‐drug therapies should be deployed in people with DRE related to different epilepsy factors are needed.

**Plain Language Summary:**

Neuromodulation can be a very helpful treatment in people who have seizures that do not respond to medications. The most widely utilized neuromodulation therapy is vagus nerve stimulation (VNS). We present data from a large, global study to show that people use an average of seven anti‐seizure medications before attempting VNS Therapy and that it takes about 10 years for people to get their first VNS implant. We advocate for clearer treatment guidelines on how and when to consider VNS Therapy in people with seizures that are resistant to medication.

**Key Points**

- Delays are common for DRE patients to receive VNS Therapy.
- Individuals with combined focal and generalized epilepsy were implanted with VNS Therapy earlier than those with focal epilepsy.
- Patients with genetic and immune epilepsies were more likely to receive VNS earlier than those with an unknown etiology.

## 1 INTRODUCTION

Drug‐resistant epilepsy (DRE) is defined by the International League Against Epilepsy as the failure to achieve seizure freedom (no seizure for at least 12 months) after adequate trials of two tolerated and appropriately chosen anti‐seizure medications (ASMs), either as monotherapy or in combination.^1^ Over 30% of individuals are unable to achieve remission with ASMs alone.^2^ In such instances, non‐drug therapies, including resective surgery, neurostimulation devices and dietary therapy, are viable considerations.^3^

Vagus Nerve Stimulation (VNS) Therapy™ has been an approved neurostimulation therapy for over 25 years (Europe 1994; US 1997). It is used as an adjunctive treatment of DRE. The VNS Therapy system consists of an implantable pulse generator (IPG) that delivers electrical stimulation and a lead placed around the vagus nerve (usually on the left side). In recent years, VNS generators also include seizure detection and nerve stimulation features. Stimulation is delivered in different modes. In standard mode, stimulation is delivered at regular intervals.^4^ The magnet mode may be triggered by the person with epilepsy or a caregiver.^5^ Use of the magnet provides additional stimulation with the intention to abort a seizure or to test battery status. Earlier VNS models dwelt more on refining battery life, size and usability issues. More recent “responsive” generators such as AspireSR™ and Sentiva™/SentiveDuo™ include an option for “Autostim Mode” which functions in conjunction with the previously described settings. As heart rate may increase suddenly during a seizure,^6^, ^7^ auto‐stimulation is activated when the relative heart rate of the individual increases above a pre‐defined threshold (for instance, ≥20% of baseline). In the newest VNS model, there are additional features that allow automatic scheduled increases of the output current (Scheduled Programming), reducing the need for hospital visits to have VNS settings adjusted. The newer models such as SenTiva and SenTivaDuo (US market approval 2017 and 2022, respectively) are programmed to deliver varied stimulation parameters at different times during a 24‐hour period (Day/Night Programming).^8^ This allows clinicians to customize stimulations for each individual thereby enabling precise targeted delivery of the stimulation and improving tolerability.

While there is accumulated evidence on outcomes of people treated with VNS stimulation therapy, previous studies have had small sample sizes (less than 100 participants) and were terminated early owing to lack of participant enrolment or investigated older versions of implanted devices (non‐responsive VNS devices). In addition, several of these studies report limited positive VNS response outcome while focusing primarily on a specific seizure type, such as focal seizures, or have reasoned that any positive impact was due to a combined adjustment to ASMs and VNS.^9^, ^10^, ^11^, ^12^, ^13^, ^14^ Given the recent technological advances in VNS, it is important to evaluate effectiveness and adverse events of the newer responsive generators.

The **C**omprehensive **O**utcomes **R**egistry in **S**ubjects with **E**pilepsy **T**reated with **V**agus **N**erve **S**timulation Therapy™ (CORE‐VNS; NCT03529045) has been established for this purpose. CORE‐VNS is a multicentered post‐market registry collecting data on the clinical outcomes among children and adults with DRE treated with VNS Therapy. The registry aims to provide real‐world experience to guide people with epilepsy, clinicians, regulators and payers regarding the use of VNS Therapy. Here, we report the baseline characteristics of the individuals enrolled in the registry. In addition, given that duration of epilepsy was often identified as a predictor of positive outcome in individuals implanted with VNS Therapy devices,^15^, ^16^, ^17^, ^18^, ^19^, ^20^, ^21^ we analyzed clinical characteristics with a hypothesis that certain baseline characteristics will be associated with timing of implantation.

## 2 METHODS

### 2.1 Study design

Participants were recruited into the CORE‐VNS from May 2018 to June 2021. Details of the study design and the study protocol have been previously reported.^8^ Briefly, participants with DRE implanted with VNS for the first time or who underwent replacement of the implanted pulse generator (IPG) device were eligible for recruitment. Participants were recruited before device implantation and followed for a minimum of 36 months, and up to 60 months, after VNS implantation. Data were collected at baseline before implantation and at 3, 6, 12, 24 and 36 months and if applicable, at 48 and 60 months. Various psychosocial measures such as Quality of life Questionnaire and Pittsburgh Sleep Quality Index Questionnaire baseline were measured at baseline and during follow up. These measures of the CORE‐VNS analyses are ongoing and beyond the scope of this paper. During the baseline visit, informed consent, demographic data, medical history, epilepsy history, seizure frequency and current treatments for epilepsy were collected. Participants also completed assessments on seizure severity, quality of life, quality of sleep and healthcare utilization. A maximum delay of 45 days was allowed between baseline assessment and device implantation.^8^ The number of participants implanted with the more recent models of generator was tracked. Use of the new device features was also monitored.

### 2.2 Participant eligibility and enrolment

Details of the eligibility criteria have been published previously.^8^ Briefly, eligible participants had to be aged at least 1 year to a maximum of 75 years, have a clinical diagnosis of DRE and be planned for VNS implantation or VNS replacement. Access to specific VNS Therapy devices was in accordance with local regulatory approvals.

To reflect real‐world practice, there was no deviation from standard clinical care and the treating clinical team decided the suitability of the participant for VNS implantation/replacement and offered the participant the choice to take part in the study.

### 2.3 Protocol approvals and informed consent

The use of participant's information from the CORE‐VNS registry lconforms to the ethical principles that have their origin in the Declaration of Helsinki and are consistent with Good Clinical Practice described in ISO 14155, and the applicable regulatory requirement(s). The study was approved by the institutional review boards or independent ethics committees of each study location.^8^ Written informed consent was obtained from each participant, or their legally authorized representative, before the participant entered the study. Written informed consent for scientific use of the clinically acquired data was also obtained.

### 2.4 Device information

The functionality of the implanted device was noted. For this purpose, devices with ability for auto‐stimulation, namely AspireSR™, SenTiva™ and SenTiva Duo™, are considered responsive VNS generators.^5^ The most recent models SenTiva™ and SenTiva Duo™ have enhanced features of auto‐stimulation and day/night programming capabilities. The older device models, Pulse M102, Demi‐Pulse M103, and their dual pin versions are defined as unresponsive as they do not deliver auto‐stimulation.^8^

### 2.5 Statistical analysis

The statistical analysis plan for the full CORE‐VNS prospective registry has been previously described.^8^ The present analysis has two parts. First, we describe the baseline characteristics of participants in the modified enrolled (mENR) population, defined as those who had provided written informed consent and met all eligibility criteria. In addition, the modified safety population (mSAF) is a subset of mENR and defined as all mENR participants who underwent a VNS Therapy implant procedure regardless of whether it was successful or unsuccessful. Second, a post hoc analysis was performed on participants receiving VNS Therapy for the first time to identify any association between time from diagnosis to first implantation and baseline characteristics. A Cox Proportional Hazards Model within SAS version 9.4 was used in this analysis performed on first implanted subjects. The following eight covariates were considered in the model: (1) the number of seizures at baseline as well as the sum of seizures at baseline regardless of seizure type, (2) the type of epilepsy, (3) sex, (4) number of prior failed ASMs, (5) previous brain or epilepsy surgery: (yes, no), (6) cognitive status: normal/minimal impairment; moderate/severe impairment, (7) etiology of epilepsy (genetic, immune, infectious, metabolic, structural, unknown) and (8) type of seizures (as identified in Table 1).

**TABLE 1:** Seizure prioritization scheme.

Seizure types	Priority
Focal aware (simple partial), motor	7
Focal aware (simple partial), non‐motor	8
Focal impaired awareness (complex partial), motor	6
Focal impaired awareness (complex partial), non‐motor	6
Focal onset, unknown awareness, motor	7
Focal onset, unknown awareness, non‐motor	8
Focal onset, unknown awareness, unknown motor	8
Focal to bilateral tonic–clonic (complex partial with secondarily generalized tonic–clonic)	3.2
Generalized atonic	2
Generalized myoclonic	5
Generalized onset, motor, clonic	3
Generalized onset, motor, epileptic spasms	3
Generalized onset, motor, tonic	3
Generalized onset, motor, unknown	3
Generalized tonic–clonic	1
Unclassified	8
Unknown onset, motor	8
Unknown onset, non‐motor	8
Generalized onset, non‐motor (absence), eyelid myoclonia	4
Non‐motor (absence) atypical	4
Non‐motor (absence) typical	4

If the participant had multiple seizure types at baseline, then for the purposes of statistical analysis, the seizure type with highest “priority” was included. Table 1 describes the seizure prioritization scheme applied in this study. Seizure types were assigned priorities in groups according to their perceived severity by authors and investigators, with 1 indicating the highest priority and 8 the lowest. For example, a person with focal aware‐motor seizures and focal to bilateral tonic–clonic seizures was automatically assigned only for the latter, which is perceived to have a greater risk of adverse impact for the individual. A point to note here is that this scheme does not consider the number of seizures per type.

Statistical analysis was performed using SAS (version 9.4). *p*‐values <0.05 were considered statistically significant.

## 3 RESULTS

### 3.1 Participant characteristics

**FIGURE 1:**
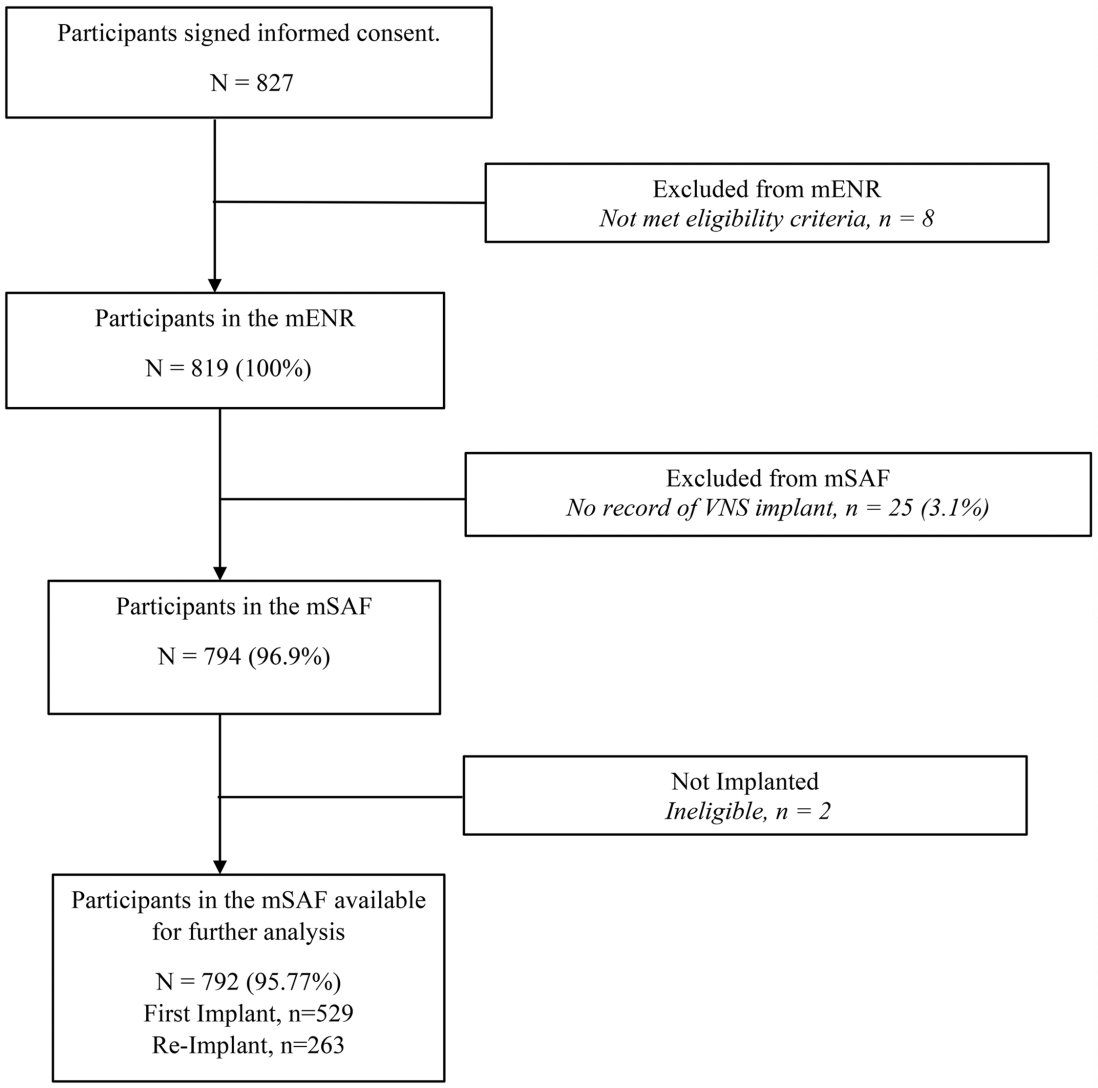
Analysis populations of all participants signing informed consent. Modified enrolled population (mENR) was defined as all participants who had provided written informed consent and met all eligibility criteria. Modified safety population (mSAF) is a subset of mENR and is defined as all mENR participants who went through a VNS Therapy implant procedure (successful and unsuccessful). Percentages are calculated on number of participants in the mENR.

Details of the participants' demographic information and numbers in each analysis set are provided as a schematic representation in Figure 1. A total of 827 participants were recruited, of whom eight were excluded due to ineligibility, leaving 819 participants who formed the mENR population. These participants were enrolled across 60 centers in 15 countries. Table 2 shows the demographic details of the mENR population. The proportions of male and female were similar. The average age at recruitment was 26.3 ± 16.5 years (range 1–75 years), with 308 (37.6%) individuals younger than 18 years and 175 (21.4%) below 12 years of age.

**TABLE 2:** Demographics and baseline characteristics of the modified enrolled population.

	mENR *N* = 819
Age at time of obtaining consent (years)	
*N*	819
Mean (SD)	26.3 (16.5)
Median	24.0
Min, max	1, 75
Age group at time of obtaining consent (years)	
<4	29 (3.5%)
4–11	146 (17.8%)
12–<18	133 (16.2%)
≥18	511 (62.4%)
Sex	
Female	403 (49.2%)
Male	416 (50.8%)

Modified Enrolled population (mENR) was defined as all participants with a signed and dated informed consent and met all eligibility criteria. Percentages are calculated on number of participants in the mENR.

Abbreviations: Max, maximum; Min, minimum; SD, standard deviation.

Table 3 summarizes baseline clinical characteristics. Almost half of the participants (47.7%) had focal epilepsy, 16.1% generalized epilepsy and 34.2% combined focal and generalized epilepsy. The type of epilepsy was unknown in a small proportion of participants (1.7%). Identified causes included structural (33.3%), genetic (17.0%) and infectious (6.2%) etiologies. In 40.9% of the participants, the etiology of epilepsy was unknown. As described in the supplemental table (Table S1), the most frequently reported epilepsy syndromes were Lennox Gastaut syndrome (*n* = 106; 12.9%), tuberous sclerosis (*n* = 32; 3.9%), infantile spasms/West syndrome (*n* = 18; 2.2%), juvenile myoclonic epilepsy (*n* = 13; 1.6%) and Dravet syndrome (*n* = 13; 1.6%).

**TABLE 3:** Epilepsy and seizure history of the modified enrolled population.

	mENR *N* = 819
Age at epilepsy diagnosis (years)	
*N*	817
Mean (SD)	8.83 (10.43)
Median	6.00
Min, max	0.5, 62.0
Age group at epilepsy diagnosis (years)	
<4	349 (42.6%)
4–11	226 (27.6%)
12–<18	117 (14.3%)
≥18	125 (15.3%)
Epilepsy type	
Combined generalized and focal epilepsies	280 (34.2%)
Focal epilepsy	391 (47.7%)
Generalized epilepsy	132 (16.1%)
Unknown	14 (1.7%)
Epilepsy etiology	
Genetic	139 (17.0%)
Immune	15 (1.8%)
Infectious	51 (6.2%)
Metabolic	4 (0.5%)
Structural	273 (33.3%)
Unknown	335 (40.9%)
Genetic non‐syndromic epilepsy^a^	
Genetic non‐syndromic epilepsy	33 (4.0%)
Non‐syndromic epilepsy^a^	
Non‐syndromic epilepsy	185 (22.6%)
Number of prior seizure medications failed^b^	
*N*	816
Mean (SD)	7.1 (3.3)
Min, max	2.0, 20
Previous brain or epilepsy surgery excluding VNS^a^	
Missing	1 (18.7%)
No	671 (81.9%)
Yes	145 (17.7%)
Type of surgery^a^, ^c^	
Lesionectomy	46 (5.6%)
Callosotomy	33 (4.0%)
Lobar resection	30 (3.7%)

*Note*: Modified Enrolled population (mENR) was defined as all participants with a signed and dated informed consent and met all eligibility criteria. Percentages are calculated on the number of participants in mENR. In case of subcategories, the relative frequencies are calculated based on the participants in the subcategory.

Abbreviations: Max, maximum; Min, minimum; SD, standard deviation.

^a^ Source data were manually recorded in a separate document from EDC.

^b^ Three participants were not implanted and were without record of prior failed medications.

^c^ One participant could have had more than one type of surgery; therefore, the total number may exceed the number of population.

In terms of treatment, the mean number of failed ASMs was 7.1 ± 3.3 (range 2–20). 145 (17.7%) individuals had undergone a previous epilepsy surgery. Of those who had epilepsy surgery, the most common operations were lesionectomy (5.6%) followed by callosotomy (4.0%) and lobar resection (3.7%).

### 3.2 Factors associated with early first implantation

Of the mENR population, 25 (3.1%) individuals did not progress to VNS implantation due to incomplete records. An additional two participants were considered ineligible due to monitoring irregularities and/or errors in record keeping (Figure 1). The remaining 792 participants were available for further analysis. Table 4 summarizes their demographic details. The majority (729/792, 92.0%) of these participants were implanted with a responsive VNS generator which was a SenTiva™ or SenTiva Duo™ in 339 (42.8%) and AspireSR™ in 391 (49.4%).

**TABLE 4:** Demographics and baseline characteristics of the modified safety population at first implantation (mSAF).

Age at first implant (years)*	
*N*	792
Mean (SD)	26.84 (16.56)
Median	24.08
Min, max	1.0, 75.1
Age group at first implant (years)	
<4	26 (3.3%)
4–11	140 (17.7%)
12–18	134 (16.9%)
≥18	492 (62.1%)
Duration between epilepsy diagnosis and informed consent (Years) (only for first implanted participants in current study)	
*n*	529
Mean (SD)	14.42 (12.74)
Median	10.00
Min, max	0.0, 62.5

Modified Enrolled population (mENR) was defined as all participants with a signed and dated informed consent and met all eligibility criteria. The mSAF population was defined as all participants in the mENR population who underwent an implantation procedure with the VNS Therapy system, whether that procedure was successful or otherwise. Percentages are calculated on number of participants in the mENR.

Abbreviations: Max, maximum; Min, minimum; SD, standard deviation.

* Biological age at first VNS implantation for new and re‐implants participants. Date of first implant will be earlier than start of the CORE‐VNS for those included in the reimplant group.

Among the 792 participants, 529 (66.8%) received their first VNS implantation and 263 (33.2%) underwent re‐implantation. In those participants who underwent their first VNS implantation, the mean duration between epilepsy diagnosis and signing of the informed consent form was 14.42 ± 12.74 years. The median interval between the age of epilepsy diagnosis and age at first implantation was 10.33 years (range 0.28 to 63.02; interquartile range 5.51 to 20.62).

Table 5 shows the findings of univariate and multivariate analyses to identify factors associated with earlier first implantation. Participants who had a higher number of seizures at baseline received VNS sooner (hazard ratio [HR]: 1.001, 95% confidence interval [CI]: 1.000–1.001, *p* < 0.05). Multivariate analysis showed that participants with genetic (HR: 1.585, CI: 1.198–2.097, *p* < 0.05) and immune epilepsies (HR: 5.390, CI: 2.719–10.684, *p* < 0.05) were more likely to receive VNS earlier than people in whom the etiology was unknown (reference group). Similarly, individuals with generalized motor seizures (including tonic, clonic, spasms) (HR: 1.668, CI: 1.065–2.612, *p* < 0.05) and generalized absences (HR: 1.795, CI: 1.183–2.724, *p* < 0.05) received VNS implantation sooner than those with generalized tonic–clonic seizures (GTCS).

**TABLE 5:** Increased hazards ratio as a measure of early implantation in participants who received first VNS implantation (*n* = 529).

Covariates	*N*	Median or %	(Q1, Q3)	(Min, max)	Univariate^a^		Multivariate^b^	
					Hazard ratio	95% HR confidence interval	Hazard ratio	95% HR confidence interval
Sex								
Male	273	51.6%	–	–	Reference group		Reference group	
Female	256	48.4%	–	–	0.958	(0.807, 1.137)	0.904	(0.752, 1.088)
Number of seizures at baseline^c^	521	50	(11, 170)	(0, 3600)	1.001	(1.000, 1.001)*	1.001	(1.000, 1.001)*
Number of prior failed ASM during lifetime	529	6	(4, 9)	(2, 20)	0.969	(0.942, 0.998)*	0.955	(0.926, 0.984)*
Type of epilepsy								
Focal epilepsy	248	46.9%	–	–	Reference group		Reference group	
Unknown	9	1.7%	–	–	1.193	(0.613, 2.323)	1.217	(0.563, 2.63)
Combined generalized and focal epilepsies	187	35.4%	–	–	1.671	(1.376, 2.028)*	1.473	(1.105, 1.964)
Generalized epilepsy	85	16.1%	–	–	1.239	(0.967, 1.586)	1.112	(0.745, 1.661)
Epilepsy etiology								
Unknown	214	40.5%	–	–	Reference group		Reference group	
Genetic	93	17.6%	–	–	1.607	(1.258, 2.054)	1.585	(1.198, 2.097)*
Immune	9	1.7%	–	–	4.229	(2.160, 8.281)*	5.390	(2.719, 10.684)*
Infectious	31	5.9%	–	–	1.147	(0.786, 1.674)	1.027	(0.688, 1.532)
Metabolic	4	0.8%	–	–	2.585	(0.959, 6.973)	2.647	(0.965, 7.262)
Structural	178	33.7%	–	–	0.875	(0.717, 1.069)	0.851	(0.686, 1.057)
Previous brain or epilepsy surgery								
No	417	78.8%	–	–	Reference group		Reference group	
Yes	112	21.2%	–	–	0.761	(0.617, 0.94)*	0.823	(0.656, 1.033)
Cognitive status								
Normal/minimal impairment	279	52.7%	–	–	Reference group		Reference group	
Moderate/severe impairment	250	47.3%	–	–	1.279	(1.076, 1.52)*	1.162	(0.948, 1.424)
Type of seizures								
GTCS	115	22.2%			Reference group		Reference group	
Focal aware motor	19	3.7%	–	–	1.066	(0.655, 1.734)	1.544	(0.878, 2.713)
Focal aware non‐Motor	11	2.1%	–	–	1.305	(0.703, 2.425)	1.768	(0.923, 3.389)
Unknown/unclassified	3	0.6%	–	–	1.139	(0.361, 3.588)	1.175	(0.362, 3.812)
G‐Atonic	23	4.4%	–	–	1.352	(0.863, 2.116)	1.275	(0.800, 2.031)
G‐Motors	27	5.2%	–	–	1.856	(1.218, 2.831)*	1.668	(1.065, 2.612)*
Focal to bilateral	101	19.5%	–	–	0.732	(0.559, 0.959)*	1.005	(0.703, 1.437)
G‐absences	30	5.8%	–	–	1.403	(0.937, 2.100)	1.795	(1.183, 2.724)*
G‐myoclonic	11	2.1%	–	–	1.017	(0.547, 1.889)	0.733	(0.386, 1.391)
Focal impaired awareness	179	34.5%	–	–	0.882	(0.697, 1.116)	1.312	(0.915, 1.881)

Abbreviations: ASM, anti‐seizure medications; GTCS, generalized tonic–colic seizures.

^a^ Univariate: One covariate was included in the model, i.e., analyses were done separately for each covariate.

^b^ Multivariate: All covariates were included in the same model.

^c^ Generalized onset absence seizures were excluded because data were collected as categories not number. One extreme value (number of generalized epileptic spasms seizure at baseline recorded as 9999) was excluded from the analysis.

* Statistical significance at *p* < 0.05, using Cox Proportional Hazards Model.

## 4 DISCUSSION

To date, CORE‐VNS is the largest prospective registry of participants treated with VNS Therapy. The current analysis provides a “snapshot” of the characteristics of participants receiving VNS therapy on a global scale in real‐world clinical practice. Over 90% of the devices implanted deliver closed‐loop stimulation with the vast majority of all participants implanted with either a Sentiva™ or AspireSR™. In this registry, 33.2% of the individuals enrolled were undergoing a replacement of the generator, suggesting long‐term efficacy of the therapy for those individuals.

People have a broad range of epilepsy etiologies and syndromes. While most individuals (40.9%) had epilepsy of unknown etiology, participants could otherwise be categorized as having structural (33.3%), genetic (17.0%) and infectious (6.2%) etiologies. The most frequently reported epilepsy syndromes were Lennox Gastaut syndrome, tuberous sclerosis, infantile spasms/West syndrome, juvenile myoclonic epilepsy and Dravet syndrome.

Among the participants who underwent implantation for the first time, the majority were adults ≥18 years old (62.1%). These individuals had a median age of 6 years at diagnosis of epilepsy, indicating a long duration between diagnosis and referral for VNS. There is emerging evidence that the time between diagnosis and implantation has decreased. In 2004, the median duration was reported to be 22 years.^22^ By 2011 a mean duration of 19 years was reported.^11^ In 2022, the median duration had reduced to 13 years,^12^ which had further decreased to approximately 10 years in our cohort. While time to VNS implantation may be gradually decreasing, the number of failed ASMs appears to have increased from a mean of 3 to around 6 observed a decade ago^10^, ^11^ to more than 7 (range 2–20) in our cohort. This may imply that people with DRE now change ASMs more frequently than before, especially as more ASMs have become available. These observations suggest that VNS is still generally regarded as a “last resort” in the clinical algorithm for treating drug‐resistant epilepsy.

Individuals with combined focal and generalized epilepsy were implanted earlier than those with focal epilepsy alone. A potential explanation for this is that people with focal DRE are initially considered for resective surgery. Surgical work‐up can take considerable time even if the final decision is not to proceed to surgery. In those who undergo surgery, time is required to determine the potential effectiveness of operative intervention. One in six participants had undergone epilepsy surgery prior to receiving VNS Therapy. We also found that participants with genetic and immune epilepsies were more likely to receive VNS earlier than those with an unknown etiology, although these etiologies were uncommon in our cohort, in particular, immune epilepsies, making it difficult to draw firm conclusion.

We observed that participants with generalized absences and generalized motor seizures were implanted earlier than those with generalized tonic–clonic seizures. Given that generalized convulsions are associated with the greatest risk, it may be anticipated that people who continue to experience this seizure type after appropriate trials of two ASMs rapidly progress to non‐pharmacological adjunctive therapy. Our data did not, though, reflect this. The explanation for this is currently uncertain and might be related to the frequency of each seizure type. For instance, a person with infrequent generalized tonic–clonic seizures may continue to receive ASM treatment alone, while one with frequent tonic or atonic seizures may be referred sooner for VNS. Future analysis will evaluate if specific seizure types better respond to VNS Therapy to potentially enable earlier consideration of VNS implantation in these individuals.

### 4.1 Limitations

While the registry offers valuable insights, the analysis of factors associated with early implantation is exploratory. CORE‐VNS is a global all comers, post‐market registry. Therefore, despite best efforts described in the protocol,^8^ there is heterogeneity in participant selection and epilepsy care between local regions and across different countries, which are subject to regional regulatory guidelines. Finally, the age at implantation in different countries is strongly dependent on the enrolment in the registry of pediatric centers. While this lack of homogeneity can be viewed as a disadvantage, we contend that our methodology much better captures real‐life clinical practice across different countries and provides a global perspective. To reduce variability, sub‐group analysis by geographical region will be performed in the future.

## 5 CONCLUSION

Taken together, the initial results from this registry show that a large proportion of participants undergoing VNS Therapy are receiving the newer models with programmable features. This will allow prospective evaluation and direct comparison of efficacy and tolerability between the responsive and non‐responsive devices. Analysis of baseline demographics features suggests that VNS is still deployed late in the treatment algorithm of drug‐resistant epilepsy, particularly in people with focal epilepsy and possibly in those with certain etiologies or seizure types. This suggests that the timing of VNS therapy may be influenced by the variable treatment pathways for DRE associated with different epilepsy types and etiologies. Clear guidelines on when and how non‐drug therapies should be deployed in people with DRE related to different epilepsy factors are needed.

## AUTHOR CONTRIBUTIONS

All authors contributed to the study conceptualization and design, wherein PK, MB and AS further performed material preparation, original draft writing, review and editing, data curation and analyses. David A. Mays, PharmD, MBA provided general manuscript administrative and scientific oversight for the submission of this manuscript. The authors thank Shivani K. Maffi for providing medical writing and editorial support for submission of the manuscript, which was funded by LivaNova according to GPP3 guidelines. All authors contributed to the interpretation of the results, writing and reviewing the content, and all authors read and approved the final manuscript.

## CONFLICT OF INTEREST STATEMENT

LivaNova USA Inc. is the sponsor of the CORE‐VNS Registry and the manufacturer of the VNS Therapy system. AS, FF, JB, RT, RL, KK and PK or their institutions have received research grants, speaker fees and/or travel honoraria from LivaNova. MB is an employee of LivaNova and holds stock or stock options. FB, GC and LL are employees of LivaNova. PK, FF, RET, TJO, KK, JB, HG and AS are investigators of CORE‐VNS. FF, RT, TO, KK, JB, AS and HGS report no conflict of interest related to this work. The authors confirm that they have read the Journal's position on issues involved in ethical publication and affirm that this report is consistent with those guidelines.

## Supporting information


Table S1.

## Data Availability

Data are available upon request and may be limited due to privacy and ethical restrictions.
