# Supplementary material for: Baseline characteristics and predictors for early implantation of vagus nerve stimulation therapy in people with drug‐resistant epilepsy: Observations from an international prospective outcomes registry (CORE‐VNS)
Source: Epilepsia Open. 2024 Aug 24;9(5):1837–46. doi: 10.1002/epi4.13015 (PMC11450613; doi:10.1002/epi4.13015)
Supplement: Supplementary file 1 — Table S1. [file EPI4-9-1837-s001.docx]

**Supplemental Table**

| **Table S1: Epilepsy and Seizure History in the Modified Enrolled Population** | | | |
| --- | --- | --- | --- |
|  | mENR N=819 | | |
| Epilepsy Syndrome ^#^ | | | |
| Unknown | 353 |  | (43.1%) |
| Lennox Gastaut Syndrome | 106 |  | (12.9%) |
| Tuberous Sclerosis | 32 |  | (3.9%) |
| Infantile Spasms/West Syndrome | 18 |  | (2.2%) |
| Dravet Syndrome | 13 |  | (1.6%) |
| Juvenile Myoclonic Epilepsy | 13 |  | (1.6%) |
| Genetic syndromic epilepsy | 11 |  | (1.3%) |
| Childhood Absence Epilepsy | 7 |  | (0.9%) |
| Juvenile Absence Epilepsy | 7 |  | (0.9%) |
| Febrile Infection-Related Epilepsy Syndrome | 6 |  | (0.7%) |
| Continuous Spikes and Slow Waves during Slow Sleep | 5 |  | (0.6%) |
| Electrical Status Epilepticus of Slow Wave Sleep | 5 |  | (0.6%) |
| Doose-syndrome | 4 |  | (0.5%) |
| Angelmen's syndrome | 2 |  | (0.2%) |
| Atypical Rett Syndrome | 2 |  | (0.2%) |
| Jeavons Syndrome | 2 |  | (0.2%) |
| PCDH-19 related epilepsy | 2 |  | (0.2%) |
| Sturge Weber syndrome | 2 |  | (0.2%) |
| ADNFLE | 1 |  | (0.1%) |
| Alternating Hemiplegia of Childhood | 1 |  | (0.1%) |
| Early Infantile epileptic encephalopathy | 1 |  | (0.1%) |
| Epilepsia Partialis Continua | 1 |  | (0.1%) |
| GRIN2B Syndrome | 1 |  | (0.1%) |
| Genetic Generalized Epilepsy | 1 |  | (0.1%) |
| Glut 1 deficiency syndrome | 1 |  | (0.1%) |
| HHE syndrome | 1 |  | (0.1%) |
| Idiopathic Photosensitive Occipital Epilepsy | 1 |  | (0.1%) |
| Miller Dieker Syndrome | 1 |  | (0.1%) |
| NORSE | 1 |  | (0.1%) |
| Rasmussens Syndrome | 1 |  | (0.1%) |
| Rett Syndrome | 1 |  | (0.1%) |
| Sleep-related hypermotor epilepsy | 1 |  | (0.1%) |
| Temporal Lobe Epilepsy with hippocampal sclerosis | 1 |  | (0.1%) |
| Unverricht Lundborg disease | 1 |  | (0.1%) |
|  | | | |
|  | | | |
| ADNFLE: Autosomal dominant nocturnal frontal lobe epilepsy; HHE: Hemi convulsion-hemiplegia-epilepsy; NORSE: New-onset refractory status epilepticus.  Modified Enrolled population (mENR) was defined as all participants with a signed and dated informed consent and met all eligibility criteria.  Percentages are calculated on number of participants in the mENR. In case of subcategories, the relative frequencies are calculated based on the participants in the subcategory. ^#^ Source data were manually recorded in a separate document from EDC.  If age at epilepsy diagnosis was recoded as “<1”, then it was analysed as 0.5 year. | | | |
